# The Rat Homolog of the Schizophrenia Susceptibility Gene *ZNF804A* Is Highly Expressed during Brain Development, Particularly in Growth Cones

**DOI:** 10.1371/journal.pone.0132456

**Published:** 2015-07-06

**Authors:** Katja Hvid Hinna, Karen Rich, Åsa Fex-Svenningsen, Eirikur Benedikz

**Affiliations:** Neurobiology Research, Institute of Molecular Medicine, University of Southern Denmark, Odense, Denmark; University of Modena and Reggio Emilia, ITALY

## Abstract

A single nucleotide polymorphism in the *ZNF804A* gene, *rs1344706*, is associated with schizophrenia. The polymorphism has been suggested to alter fetal expression of *ZNF804A*. It has also been reported to be associated with altered cortical functioning and neural connectivity in the brain. Since developmental mechanisms are suggested in the pathophysiology for schizophrenia, expression of *Zfp804*A, the rat homolog of *ZNF804A*, was investigated in the developing rat brain. We found that expression of *Zfp804A* in most brain regions is developmentally regulated and peaks around birth, where after it decreases towards adult levels. This time point is developmentally the equivalent to the second trimester of fetal development in humans. An exception to this expression pattern is the hippocampus where the expression of *Zfp804A* appears to increase again in the adult brain. Using laser capture and quantitative PCR we found that *Zfp804A* mRNA expression in the adult rat hippocampus is highest in the CA1 sub region, where the overall firing rates of neurons is higher than in the CA3 region. In cultured cortical neurons *Zfp804A* mRNA expression peaked at day 4 and then decreased. The ZFP804A protein expression was therefore investigated with immunochemistry in such cultures. Interestingly, before day 4, the protein is mostly found in the perinuclear region of the cell but at day 4, ZFP804A was instead found throughout the cell and particularly in the growth cones. In conclusion we demonstrate that *Zfp804A* increases in the rat brain at the time of birth, coinciding with neuronal differentiation. We also show that ZFP804A is localized to growth cones of growing neurites. These data implicate ZFP804A in growth cone function and neurite elongation. The polymorphism *rs1344706* lowers expression of *ZNF804A* during prenatal brain development. This may affect ZNF804A’s role in cone function and neurite elongation leading to synaptic deficits and altered neural connectivity.

## Introduction

Schizophrenia is a severe psychiatric disorder characterized by delusions, hallucinations, altered cognition, emotional reactivity and disorganized behaviour [1]. Research suggests that schizophrenia is a disorder of brain development and plasticity [2] but despite extensive investigation, the etiology and pathophysiology remain poorly understood [3]. Genetics play an important role in the disease and heritability is around 80% [4]. *ZNF804A* was the first gene to achieve genome-wide significance for psychosis [1], and several studies have confirmed an association between schizophrenia and a single nucleotide polymorphism, *rs1344706*, in the *ZNF804A* gene [5–9]. This polymorphism is associated with altered expression of the gene in the dorsolateral prefrontal cortex in fetal [10, 11] but not adult brain [10–12]. The function of *ZNF804A* and its role in the disease remain unknown.

*ZNF804A* is expressed in the brain [10] but the protein, which is 1209 amino acids long, has not been characterized. Analysis of the protein sequence indicates that it contains a single C2H2-type domain that is associated with the zinc-finger protein family. Proteins with this domain were originally identified as DNA-binding molecules with a role in transcription but they can also interact with other molecules including proteins [13]. Recently a new human transcript was identified in the brain [10]. This isoform lacks the first 135 amino acids of ZNF804A, which includes the zinc-finger domain suggesting another function than a role in transcription.

Interestingly the schizophrenia susceptibility genotype of *ZNF804A* is associated with altered connectivity in the dorsolateral prefrontal cortex, the hippocampus, and the amygdala. Such alterations in connectivity within and between these same brain regions have previously been associated with schizophrenia [14, 15]. In fact, the association with *ZNF804A* provides some of the first evidence of the gene’s functional involvement in brain activity. Recently, the expression of ZNF804A was knocked down in cultured neuronal progenitor cells. The decreased expression of *ZNF804A* led to an altered expression of genes involved in cell adhesion, suggesting a role for ZNF804A in neural migration, neurite outgrowth and synapse formation [16].

To better understand the function of *ZNF804A* in the developing nervous system we investigated the expression of *Zfp804*A, the rat homolog of *ZNF804A*, in different brain regions and in cultured cortical neurons. We found that *Zfp804A* expression in the brain is developmentally regulated. Furthermore, in primary cortical neurons, ZFP804A is highly expressed in growth cones. These data further implicate ZNF804A in development and neurite outgrowth.

## Materials and Methods

### Animal tissue

Brain tissue was provided by the authorized central animal facility of the University of Southern Denmark (J.nr. 2013-15-2937-00012, Danish Animal Experiments Inspectorate).

### Ethics statement

The Sprague-Dawley rats, housed at the central animal facility, University of Southern Denmark, were euthanized by carbon dioxide inhalation in according Danish and European legislation by authorized staff. Approval of the protocol was not required, because housing and handling procedures, as well as euthanasia, are well-standardized protocols conceived and constantly monitored by the Animal Research Ethics Committee, Denmark. The rats were euthanized for the specific purposes of this study and no experimental procedures were performed on the rats prior to euthanasia.

### Laser Capture micro-dissection

Brains from adult rats were removed under RNase free conditions and immediately frozen. The frozen brains were cut into coronal sections, 30 μm thick and mounted on PET-folio slides (Leica). The slides were fixed, washed and stained with either Histogene LCM frozen sections staining kit (Life Technologies #KIT0401) in accordance with the manufacturer’s protocol, or toluidine blue. Within the hippocampal region cornu ammonis area 1 (CA1), CA3 and the dentate gyrus (DG) were micro-dissected from the slides by the use of a laser (Olympus, Leica LMD 6000). The micro-dissected tissue was stored in RA1 lysis buffer (Macherey-Nagel, NucleoSpin RNA XS # 740902.50) and later the RNA was purified. Laser Capture Micro-dissection (LCM) was conducted on tissue sections from three different rats and a total of 141 samples were collected. Samples were pooled in groups of 7–9 sections derived from both the dorsal and ventral part of the hippocampus.

### Quantitative PCR

RNA was purified from brain tissue and culture cortical neurons using Nucleospin RNA II (Macherey-Nagel) in accordance with the manufacturer’s protocol. The quantity and quality of the RNA was determined on a Nanodrop 2000C spectrophotometer (Thermo Scientific) and on 2.2% agarose gels using the FlashGel System (LONZA). 30 ng total RNA was reverse transcribed using the High-Capacity cDNA Reverse Transcription Kit (Applied Biosystems) and random primers in accordance with manufacturers protocol.

qPCR was performed in a CFX Connect Real-Time system Thermal Cycler (Bio Rad) using 5 μl SsoFast EVAgreen Supermix (Bio Rad), 5 pmol primer mix against either *Zfp804A* or the housekeeping gene glyceraldehyde-3-phosphate dehydrogenase (*Gapdh*). The primers for Zfp804A target sequences in exon 1. *Gapdh* was chosen because it has been shown to be stably expressed in brain tissue (S1 Fig) [17, 18]. All qPCR primer sets were tested for specificity and efficiency, and optimized in accordance with the MIQE guidelines [19]. The specific primer-sequences (S1 Table) and the PCR programs used (S2 Table) are found in the supporting information.

The qPCR experiments were performed in triplicates with negative controls (no template or no reverse transcription) for each primer pair on each plate with a standard curve showing PCR efficiency between 90–110%. In addition to melting point curves, product lengths where analyzed on agarose gels using the FlashGel System (LONZA) to assure specificity.

Parallel assays for each sample were performed using *Gapdh* for normalization. Standard curves were prepared for each target using serial dilutions of a single sample. All experiments were repeated several times and relative gene expression was determined using the “efficiency calibrated mathematical method for the relative-expression ratio in real-time PCR” described in Pfaffl, 2001 [20].

### RNA quality

In this type of gene expression study, the quality of the RNA used is crucial for obtaining reliable data. The RNA quality for each sample was assessed by a spectrophotometer, and selected samples were run on a RNA Flash gel to assess the integrity of the 28S and 18S bands. Samples with absorbance measurement ratios at 260/280 nm ≤1.85 and ≤1.70 for 230/260 nm were excluded. RNA samples that showed a 230/260 nm ratio close to 1.70 were always investigated for intact 28S and 18S bands on a gel. Only samples that fulfilled the quality criteria were assessed by qPCR.

### Primary neurons

Primary neuronal cell cultures were prepared from the cortex of Sprague Dawley rat embryos on day 18 of gestation [21, 22]. Briefly, pregnant females were euthanized with CO_2_ and subjected to caesarean section in order to remove the fetuses. The uterus was surgically removed from the adult rat and placed in a 100mm petri dish containing cold Leibovitsz’s (L15) medium (Invitrogen). The embryos were decapitated and the brains removed. The cerebral cortex was isolated and the hippocampus and meninges were removed. The tissue was transferred to a tube containing L15 medium and kept on ice. The tissue was mechanically dissociated, passed through a cell strainer to remove cellular debris and centrifuged at 250 g. The cells were re-suspended in Neurobasal medium supplemented with 0.3% L-glutamine, 2% B27 and 1% penicillin/streptomycin, and plated onto poly-L-Lysine coated flasks and plates. Cells were grown in a humidified atmosphere of 95% air and 5% CO2 at 37°C for up to 4 weeks with medium renewal every 3–4 days. At different time points cells were harvested for analysis of ZNF804A mRNA by qPCR.

### Immunocytochemistry

After 2–28 days of growth primary neurons were fixed in 4% paraformaldehyde (PFA) in PBS, washed three times x 5 minutes in PBS, incubated over night at 4°C in PBS containing 10% sucrose and then frozen at -20 until used. The cells were then washed in PBS and blocked in blocking buffer (PBS pH 7.2 containing 0.25% BSA and 0.25% Triton-X-100) for 1hr at room temperature. The primary antibodies, anti-ZNF804A (D14, Santa Cruz) diluted 1:50 and doublecortin (Abcam) diluted 1:2000 in blocking buffer, were incubated over night at 4°C. The specificity of the anti-ZNF804A antibody was verified by Western blot of ZNF804A transfected HEK cells, as others have done previously [23]. Following primary antibody incubation, cultures were washed in PBS containing 0.25% Triton-X-100 and incubated with secondary antibodies (Alexa Fluor 594 anti-goat IgG and Alexa Fluor 488 anti-rabbit both diluted 1:600, Invitrogen) diluted in blocking buffer. Controls were made with only secondary antibody to avoid investigating unspecific labelling of cells. The slides were then washed twice and mounted in DTG mounting media (2.5% DABCO (Sigma-Aldrich), 50 mM Tris-HCl pH 8.0, 90% glycerol) with or without 0.375 mg/ml DAPI (Sigma-Aldrich). Culture slides were analysed at Olympus FV1000 confocal laser scanning microscope. Some cells were analyzed in three dimensions by confocal laser-scanning microscopy.

### Statistical analyses

Comparisons of relative *Zfp804A* mRNA expression between the different groups was analysed using one-way ANOVA followed by Bonferroni’s multiple comparison test.

## Results

### Expression of Zfp804A peaks around the time of birth

**Fig 1:**
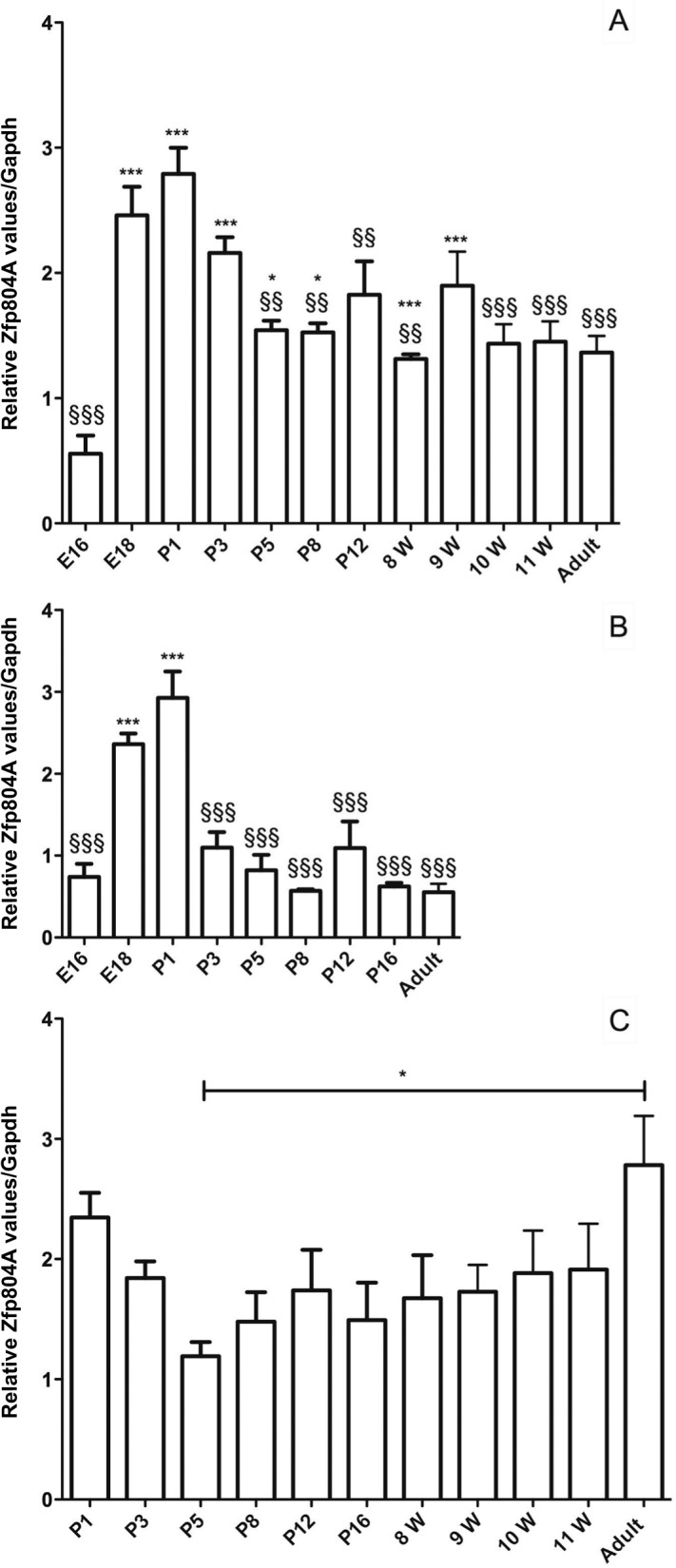
*Zfp804A* mRNA expression in the developing rat brain. (A) Relative *Zfp804A* mRNA expression in the frontal part of the rat cortex. *Zfp804A* expression is significantly increased at E18, P1 and P3 compared to the levels at E16 (***P<0.001) and after P3 the level decreases significantly compared to P1 (^§§^ *P*<0.01, ^§§§^ *P*<0.001). (B) Relative *Zfp804A* expression in the rat cerebellum. The expression of *Zfp804A* is significantly higher at E18 and P1 compared to E16 (***P<0.001) and lower at all time points compared to P1 (^§§§^ *P*<0.001) except for E18. (C) Relative *Zfp804A* expression within the rat hippocampus. Expression is significantly increased in the adult rat compared to P5 (*P<0.05). The zfp804A primer set was used and data are presented as means ± SEM. *Gapdh* was used as a reference gene and n ≥ 4

The mRNA levels of *Zfp804A* were investigated in different rat brain regions from animals aged E16 to adult to determine if the expression of this protein is stable or changes over time. At E16, the levels of *Zfp804A* mRNA were low in the frontal cortex and cerebellum. Shortly before birth, at E18, the levels increase 3–5 fold, peaking at postnatal day 1 (P1). The mRNA levels dropped again at postnatal day 5 (P5) in the frontal cortex and P3 in cerebellum (Fig 1A and 1B) to levels similar to those found in adult brain. The levels of *Zfp804A* mRNA are thus significantly increased in E18, P1 and P3 frontal cortex compared to the levels at E16 (*P*<0.001). In the cerebellum the levels were significantly higher at E18 and P1 compared to E16 (*P*<0.001). In both the frontal cortex and cerebellum, the levels of *Zfp804A* mRNA were significantly lower a few days after birth, compared to the peak values at E18 and P1 (*P*<0.01).

### Expression of *Zfp804A* is highest in the hippocampal CA1 subregion

**Fig 2:**
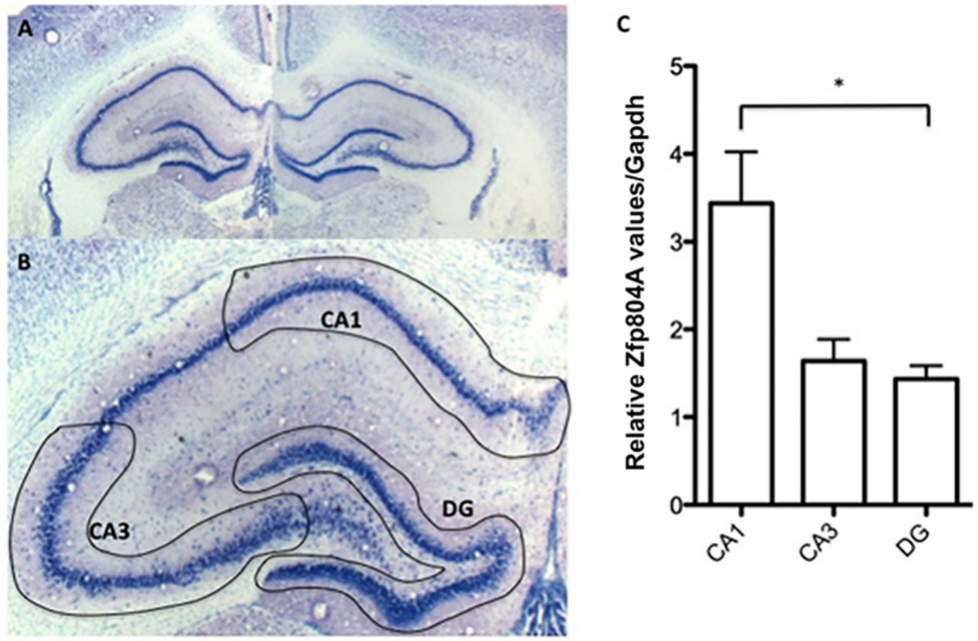
*Zfp804A* mRNA expression in the adult rat hippocampus. (A) Adult rat section stained with toluidine. (B) Schematic illustration of the dissected regions of the hippocampal sub-regions CA1, CA3 and DG. (C) Relative *Zfp804A* expression in the three sub-regions of a rat hippocampus. The zfp804A primer set was used and data is presented as means ± SEM. *Gapdh* was used as a reference gene and n ≥ 4. There is a significant difference between CA1 and DG (*P < 0.05).

The levels of *Zfp804A* mRNA did not change as much in the hippocampus as it did in the frontal cortex or cerebellum (Fig 1C). However, the same pattern can be seen, with high levels at P1 and a decrease at P5 to the lowest level of the time points investigated. Interestingly the highest levels of *Zfp804A* detected in the hippocampus were found in the adult samples. In fact these values are significantly higher than at P5 (*P*<0.05). We therefore investigated the expression levels of *Zfp804A* in animals aged 8–11 weeks, which corresponds to adolescence in the rats but the expression did not reach adult levels during this period. Since the dorsal and ventral parts of the hippocampus are functionally distinct, gene expression may differ [24]. The dorsal, ventral and medial part of the adult hippocampus were dissected, and the mRNA levels determined in the different parts. The results showed no difference in expression. We also investigated mRNA expression within the adult hippocampus using LMC (Fig 2). The results showed that the expression of *Zfp804A* was significantly higher in the CA1 subfield compared to that in DG (*P* < 0.05) (Fig 2C). The levels in CA3 were similar to that in DG, but when compared to the CA1, the difference did not reach significance.

### Expression of Zfp804A peaks at day 4 in cultured primary neurons

**Fig 3:**
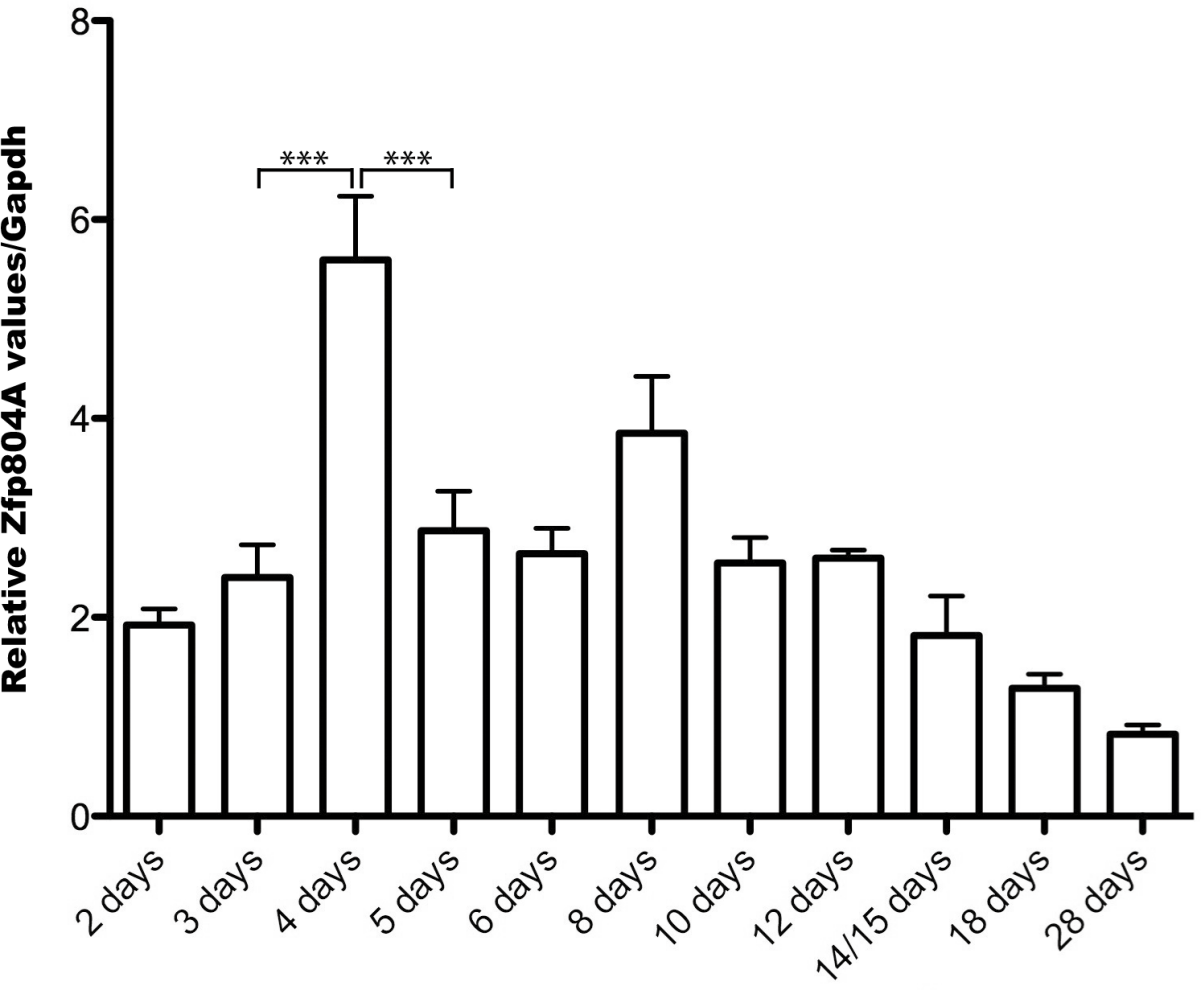
*Zfp804A* mRNA expression in cultured cortical neurons. The relative *Zfp804A* mRNA expression was determined in primary cortical neurons at different days *in-vitro*. The levels at day 4 were significantly increased (***P<0.001). The zfp804A primer set was used and data is presented as means ± SEM. *Gapdh* was used as a reference gene and n ≥ 4.

To determine if the *Zfp804A* expression peak we detected *in-vivo* could be modelled in a culture system, we prepared primary neuronal cortical cultures from the cerebral cortex from E18 rat embryos and isolated mRNA from cells at different days in vitro (DIV). We did indeed find a significant peak in the expression of *Zfp804A* (p< 0.001) in the primary neurons at 4 DIV (Fig 3). Following the peak, the levels again dropped significantly and remained stable until 12 DIV when the levels decreased further.

### ZFP804A is highly expressed in growth cones

**Fig 4:**
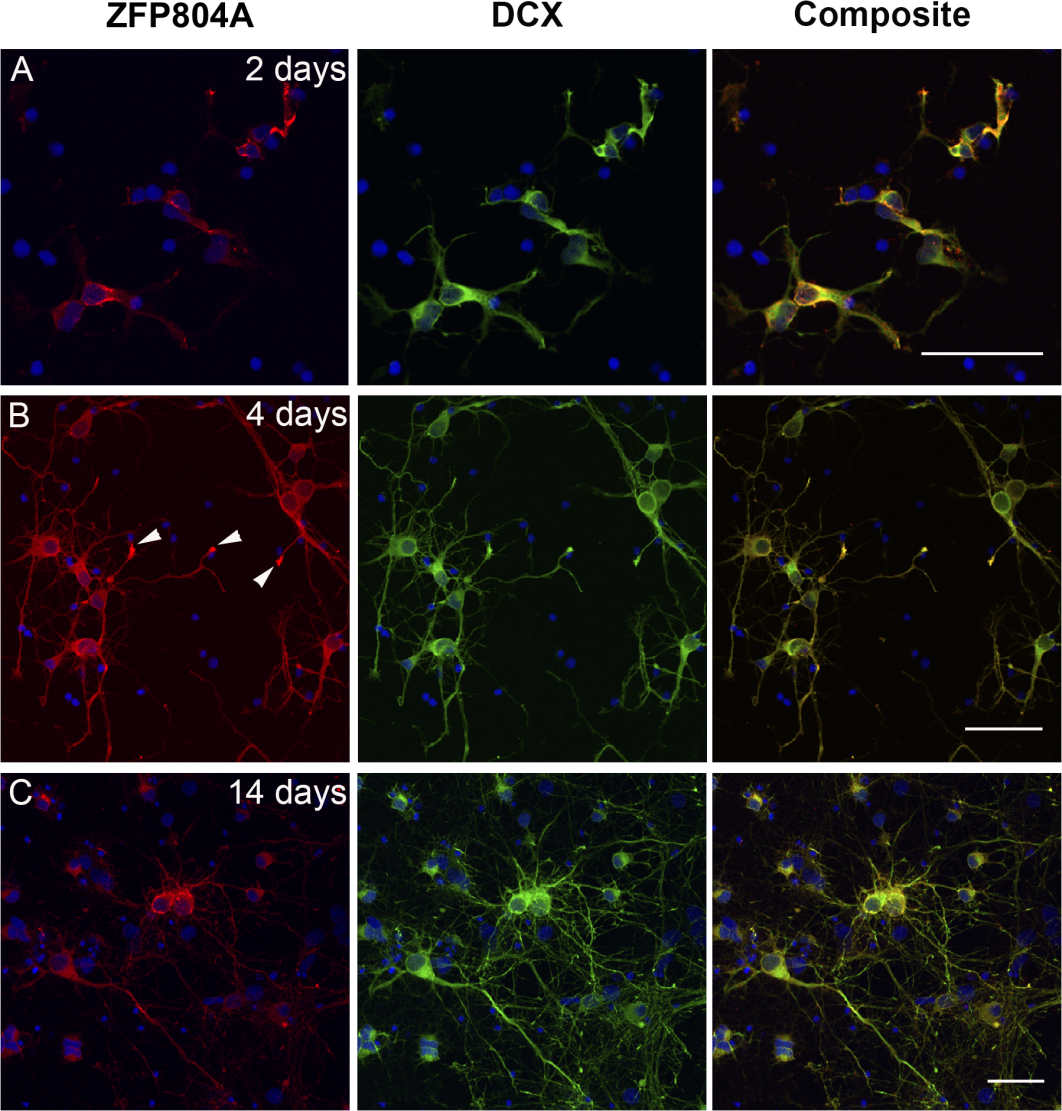
Distribution of ZFP804A in cultured primary neurons changes over time. Primary cortical neurons were immune-labelled for ZFP804A and doublecortin. (A) 2 DIV, ZFP804A is found predominantly in the cytoplasm. (B) 4 DIV, ZFP804A is found throughout the neuron, particularly in the growth cones. (C) 14 DIV, ZFP804A is redistributed and is again found mostly surrounding the nucleus. Arrow heads in (B) indicate growth cones immune-labelled for ZFP804A. Scale bar = 50 μm.

**Fig 5:**
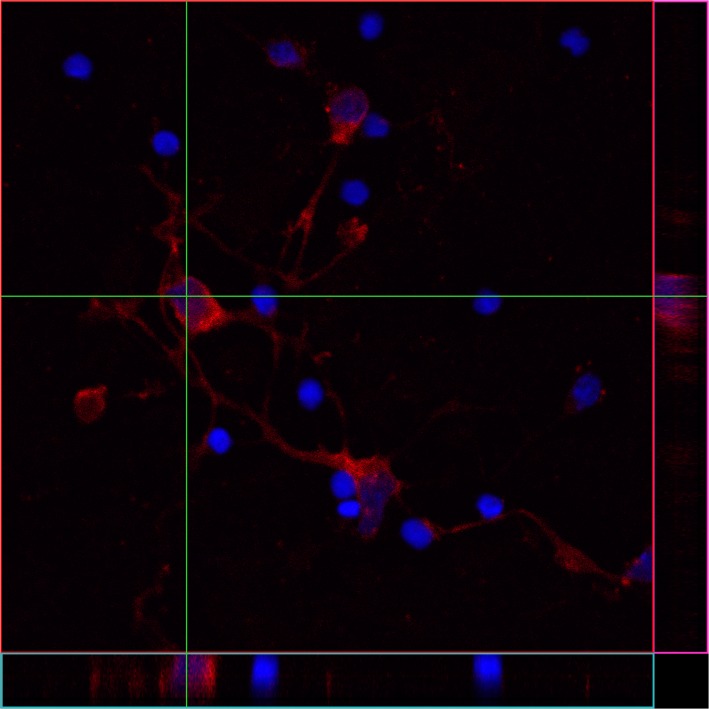
ZFP804A is found in the cytoplasm and nucleus. Confocal ortho-images of *Z*-stacks of cultured neurons show that ZFP804A is also present in the nucleus. The center image is the *X-Y* view, the images below and right are *X-Z* and Y-Z views (cross section at the green line).

To identify the cellular compartments where ZFP804A is located, cultured neurons were immune-labelled at different time points. Interestingly, the ZFP804A protein distribution changed over time with development. At 2 DIV ZFP804A was mostly found in the cytoplasm surrounding the nucleus with weaker staining in the neurites (Fig 4A). The few growth cones that are seen at 2 DIV are intensively labelled for ZFP804A. At day 4, when the mRNA levels have increased significantly, the immuno-labelling is very strong throughout the whole neuron, particularly in the growth cones (arrows Fig 4B). At 6 DIV ZFP804A labelling is still strong throughout the neuron but from 8 DIV, it clearly decreases. As the amount of ZFP804A decreased, the protein is redistributed and instead found mostly in the cytoplasm (Fig 4C). The number of growth cones in the older cortical neuronal cultures is low, but those that were found are still intensely labelled for ZFP804A. There have been conflicting reports concerning the subcellular location of ZFP804A. Although ZFP804A is predominantly in the cytoplasm of primary cortical neurons, confocal ortho-images of *Z*-stacks illustrate that it is also present in nucleus (Fig 5).

## Discussion

Today schizophrenia is increasingly viewed as a disorder of neurodevelopment. This is based on both the fact that changes in brain structure and cognitive function are associated with schizophrenia and that several genetic association studies have identified schizophrenia risk genes that are in involved in brain development [25–28].

Here we report that *ZFP804A* mRNA expression is developmentally regulated in the cortex, hippocampus as well as the cerebellum. This pattern was not seen in the brainstem (pons and medulla) or the spinal cord (preliminary data) where *ZFP804A* was stably expressed at low levels at the same time points. A possible up-regulation within these CNS regions may occur earlier during embryonic development since they develop earlier. In the cortex, expression of *ZFP804A* increases dramatically in the rat brain just before birth and stayed high until about postnatal day 3. This up-regulation coincides with the period when neuronal migration ends and neuronal differentiation and maturation begins in the developing rat cortex. In the human cerebral cortex these processes start earlier; during the second trimester. It has recently been suggested that *ZNF804A* expression in the human brain peaks during this period of fetal development (10, 24). Furthermore, the polymorphism, *rs1344706*, in the *ZNF804A* gene, has a significant effect on *ZNF804A* allelic expression in second-trimester where it is associated with reduced *ZNF804A* expression [11]. A recent study, focusing on the dorsolateral prefrontal cortex, confirmed this finding but showed that the reduction is due to a lower expression of the truncated transcript and not the full-length transcript (10). These data thus suggest that the expression of ZNF804A is highest under the period of embryonic development when neuronal differentiation occurs in the CNS. This is in accord with additional findings showing that knocking down *ZNF804A* in human neural stem cells decreases the expression of genes involved in cell adhesion [16], while over-expression of ZNF804A in HEK293 cells up-regulated genes associated with TGF-β signaling which plays a role in cell growth and differentiation [29]. This means that both over-expression and knock-down of ZNF804A alters the expression of proteins involved in neural migration, neurite outgrowth and synapse formation.

To further investigate ZFP804A localization during neural differentiation primary cortical neurons were investigated. Here we found a similar peak in mRNA expression (4 DIV) as previously found *in vivo*. This peak in Zfp804A expression occurs at the time when dendritic development has begun, there are numerous growth cones in the culture and the first putative synaptic boutons are formed. In the cultured neurons at 4 DIV, ZFP804A is highly expressed especially in the growth cones. At an earlier time point (2 DIV) ZFP804A is weaker and mostly localized to the cell soma and at later time points, when the number of growth cones decrease, ZFP804A is again more prominent in the cell soma. These data suggest that ZFP804A may be involved in neurite elongation or growth cone motility. When contact is reached between neurons the ZFP804A expression diminishes. This distribution in the older cultured neurons is also in agreement with previous findings concerning neuronal distribution in the human brain [10].

It has been suggested that ZNF804A is a transcription factor, but the classical transcription factors are concentrated to the nucleus and ZFP804A is not. Although it is present in the nucleus, the immunoreactivity is predominantly found in the cytosol suggesting different functions for this protein. This further supported by the fact that a truncated form of ZNF804A lacking the zinc-finger domain has been identified.

*The Zfp804A* expression in the hippocampus differed from that seen in the frontal part of the cortex and the cerebellum. The levels in the adult hippocampus were higher and similar to the levels in new-born rats. This may suggest that ZFP804A is involved hippocampal function. A previous study has furthermore implicated the *rs1344706* genotype in the modulation of connections between the dorsolateral prefrontal cortex (DLPFC) and the hippocampus [30]. For this reason we further investigated the different parts of the hippocampus but found no difference in *Zfp804A* expression between the dorsal and ventral parts. However, investigating the different sub-regions of the hippocampus, we found that the levels of *Zfp804A* mRNA in CA1 were higher than in CA3 or DG. This is also consistent with the data from *in situ* hybridization shown in the Allan mouse brain atlas [31]. It has previously been reported that the overall firing rates of CA1 neurons are significantly higher than those of CA3 cells [32]. This might explain why the activity regulated cytoskeleton-associated protein (Arc) expression is higher in CA1 than CA3 [33]. Arc is an indicator of cellular activity and expressed during neuronal activation associated with information processing and is essential for synaptic plasticity [34–36]. The differential expression pattern of *Zfp804A* in the hippocampus may suggest that its expression is regulated by neuronal activity.

In conclusion, we report that *Zfp804A* increases in the CNS at the time of birth coinciding with neuronal differentiation in the rat. Furthermore, we show that ZFP804A is localized to growth cones and that these data implicate ZFP804A in growth cone function and neurite elongation.The SNP *rs1344706* within *ZNF804A* is associated with lower expression during prenatal brain development [10, 11] and it is plausible that this may affect ZNF804A’s role in cone function and neurite elongation leading to synaptic deficits and altered neural connectivity. It has in fact been suggested that this SNP affects cortical functioning [37, 38] and neural connectivity [30] in adults.

## Supporting Information

S1 Fig Levels of *Gapdh*/ng mRNA in brain tissue from animals (n ≥ 8) of different ages. (DOCX)

S1 Table Quantitative PCR primer sequences. (DOCX)

S2 Table qPCR programs. (DOCX)
