# Supplementary figures and images for: The Rat Homolog of the Schizophrenia Susceptibility Gene ZNF804A Is Highly Expressed during Brain Development, Particularly in Growth Cones
Source: PLoS One. 2015 Jul 6;10(7):e0132456. doi: 10.1371/journal.pone.0132456 (PMC4493006; doi:10.1371/journal.pone.0132456)

**Figure S1. Levels of *Gapdh*/ng mRNA in brain tissue from animals (n ≥ 8) of different ages**


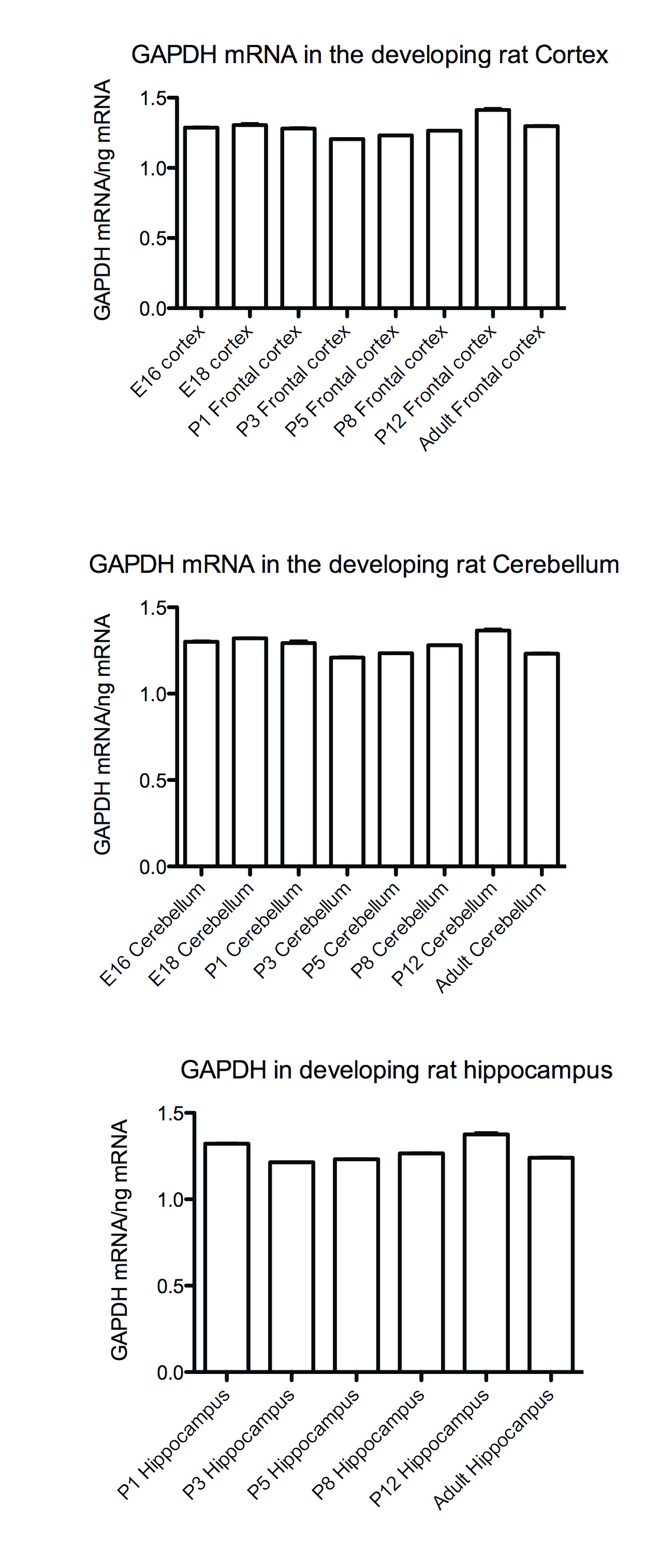

Supplement: S1 Fig — (DOCX) [file pone.0132456.s001.docx]
