# Supplementary material for: The Rat Homolog of the Schizophrenia Susceptibility Gene ZNF804A Is Highly Expressed during Brain Development, Particularly in Growth Cones
Source: PLoS One. 2015 Jul 6;10(7):e0132456. doi: 10.1371/journal.pone.0132456 (PMC4493006; doi:10.1371/journal.pone.0132456)
Supplement: S1 Table — (DOCX) [file pone.0132456.s002.docx]

## Supplementary table

## Table S1. Quantitative PCR primer sequences

| **Name** | **Sequence** | **Amplicon Length** |
| --- | --- | --- |
| Zfp804Af | 5´-CTCCACGCATCTCAGCAAC-´3 | 19 bp |
| Zfp804Ar | 5´-GCCCCGGAAAACTCCCTTG-´3 | 19 bp |
| rGapdhf | 5´- GAACATCATCCCTGAATCCA-´3 | 20bp |
| rGapdhr | 5´- CCAGTGAGCTTCCCGTTC-´3 | 18bp |
