# Supplementary material for: The Rat Homolog of the Schizophrenia Susceptibility Gene ZNF804A Is Highly Expressed during Brain Development, Particularly in Growth Cones
Source: PLoS One. 2015 Jul 6;10(7):e0132456. doi: 10.1371/journal.pone.0132456 (PMC4493006; doi:10.1371/journal.pone.0132456)
Supplement: S2 Table — (DOCX) [file pone.0132456.s003.docx]

## Supplementary table

## Table S2. qPCR programs

rat *Zfp804A* (Zfp804A primer set)

| Step | Temperature | Time |
| --- | --- | --- |
| 1 | 98°C | 2 min |
| 2 | 98°C | 5 sec |
| 3 | 62°C | 10 sec |
| 4 | Plate read. Go to step 2 for 39 times | |
| 5 | Melting curve. 0,5°C / 5 sec (65-95°C) | |

rat *Gapdh* (rGapdh primer set)

| Step | Temperature | Time |
| --- | --- | --- |
| 1 | 98°C | 2 min |
| 2 | 98°C | 5 sec |
| 3 | 62°C | 5 sec |
| 4 | Plate read. Go to step 2 for 39 times | |
| 5 | Melting curve. 0,5°C / 5 sec (65-95°C) | |
